# Supplementary material for: The Effectiveness of Mental Health First Aid Training among Undergraduate Students in Switzerland: A Randomized Control Trial
Source: Int J Environ Res Public Health. 2023 Jan 11;20(2):1303. doi: 10.3390/ijerph20021303 (PMC9859566; doi:10.3390/ijerph20021303)
Supplement: Supplementary file 1 [file ijerph-20-01303-s001.zip › ijerph-2132693-SI.pdf]

Supplement

Table S1 Model based mean or frequency of knowledge, attitude, and behavior in Control and Intervention groups at three-time points.

Variables	**T0 (N=107)**		**T1 (N=78)**		**T2 (N=71)**	
	**Control mean (SE)**	**Intervention mean (SE)**	**Control mean (SE)**	**Intervention Mean(SE)**	**Control mean (*SE*)**	**Intervention mean (SE)**
MHFA knowledge	12.69 (0.27)	13.13 (0.24)	12.90(0.29)	14.33(0.26)	12.77 (0.32)	14.08(0.31)
Attitude: Personal stigma	16.11(0.40)	16.23(0.42)	15.87(0.41)	13.88(0.42)	16.10(0.47)	14.15(0.47)
Social distance	24.00 (0.75)	25.51 (0.58)	24.74 (0.80)	28.26 (0.90)	24.36 (0.91)	28.38 (0.82)
Behavior:						
Confidence to help	2.70 (0.12)	2.81 (0.12)	2.84 (0.13)	3.76 (0.09)	2.94 (0.13)	3.67 (0.11)
Intention to help	2.17 (0.15)	2.17(0.15)	2.78 (0.25)	3.41 (0.33)	2.74 (0.20)	2.70 (0.19)
	**T0**		**T1**		**T2**	
	**Control *n* (adj %)**	**Intervention *n* (adj %)**	**Control *n* (adj %)**	**Intervention *n* (adj %)**	**Control *n* (adj %)**	**Intervention n (adj%)**
Recognition of disorder						
Yes	30 (55.6)	25 (47.2)	16 (39.1)	17 (44.4)	20 (53.9)	16 (44.1)
No	24 (44.4)	28 (52.8)	26 (60.9)	19 (52.8)	18 (46.1)	17 (55.9)
Confidence to help						
Yes	12 (22.2)	13 (24.5)	13 (30.5)	27 (74.1)	25 (41.1)	32 (69.4)
No	42 (77.8)	40 (75.5)	29 (69.5)	9 (25.9)	13 (58.9)	1 (30.6)
First aid action						
Yes	23 (82.4)	21 (84.1)	14 (63.5)	17 (100)	10 (83.6)	15 (93.0)
No	5 (17.9)	4 (16.0)	8 (36.4)	0	2 (16.4)	1 (7.0)
